# Frailty and Its Contributory Factors in Older Adults: A Comparison of Two Asian Regions (Hong Kong and Taiwan)

**DOI:** 10.3390/ijerph14101096

**Published:** 2017-09-21

**Authors:** Ruby Yu, Wan-Chi Wu, Jason Leung, Susan C. Hu, Jean Woo

**Affiliations:** 1 Department of Medicine and Therapeutics, Faculty of Medicine, The Chinese University of Hong Kong, Shatin, Hong Kong, China; rubyyu@cuhk.edu.hk; 2 The Chinese University of Hong Kong Jockey Club Institute of Ageing, Shatin, Hong Kong, China; 3 Department of Public Health, College of Medicine, National Cheng Kung University, Tainan 701, Taiwan; idawu218@gmail.com; 4 The Chinese University of Hong Kong Jockey Club Centre for Osteoporosis Care and Control, Shatin, Hong Kong, China; jason-leung@cuhk.edu.hk

**Keywords:** frailty, frailty index, compression of morbidity, prevalence, risk factor, healthcare system

## Abstract

This study aimed to compare the prevalence of frailty across three Chinese populations: Hong Kong, Taiwan-urban and Taiwan-rural. Contributing factors to disparities in frailty were also examined. Data were derived from the Osteoporotic Fractures in Men (MrOs) and Women (MsOs) (Hong Kong) Study (*n* = 4000) and the Taiwan Longitudinal Study on Aging (*n* = 2392). Frailty was defined as an index calculated from 30 multiple deficits. The ratio of the frailty index to life expectancy at birth (FI/LE) was used as an indicator of compression of morbidity. Frailty was more prevalent in Taiwan-urban (33.1%) and Taiwan-rural (38.1%) compared to Hong Kong (16.6%, *p* < 0.05) and was higher in women (22.6–49.7%) than in men (10.5–27.5%, *p* < 0.05). The ratios of FI/LE were higher in Taiwan-urban and Taiwan-rural (both 0.27) compared to Hong Kong (0.20, *p* < 0.05). Multivariate analyses revealed that older age, being a woman and low levels of physical activity were common risk factors for frailty across the three populations. Alcohol use was inversely associated with frailty in both Hong Kong and Taiwan-urban populations, but not in Taiwan-rural. Living alone was associated with frailty in Hong Kong men, but not in Hong Kong women or Taiwanese people. For all study populations, older age and being a woman constituted the highest attributable factor. This comparison provides useful data to inform government policies.

## 1. Introduction

With the increase in life expectancy, the prevalence of multi-morbidity is expected to increase [1], resulting in increased demand for healthcare services. Many studies have examined trends in disability among older adults aiming to identify patterns of ageing [2,3] and whether there has been a compression of morbidity [4] (i.e., the increased life expectancy accompanied by a shortening of the length of morbid life) as proposed by Fries [5,6,7]. However, disability is influenced not only by physiological functioning, but also by the environment in which it takes place. Frailty, the state representing a decline in functional reserves, has emerged as a key aspect in research on population ageing. It is used as a health indicator for how well populations are aging and is commonly used in the context of the elderly facing functional disabilities, as many studies have demonstrated that frail individuals are at a high risk of becoming disabled, independent of the presence of co-morbid diseases [8,9,10,11,12,13]; thus, frailty has been suggested as a better predictor of health and well-being than the presence or absence of disease and has been used as an indicator of the compression of morbidity to predict the demands on the healthcare system [14].

Currently, there are two frameworks (approaches) that are referred to for the assessment of frailty. A popular approach as proposed by Fried et al. [8] (i.e., the phenotype approach) encompasses the assessment of five criteria based primarily on physical attributes and capabilities including poor grip strength, slow walking speed, low levels of physical activity, exhaustion and unintentional weight loss, whereas an individual is considered to be frail if he or she meets three or more of the five criteria. Another notable approach is that of Rockwood and Mitnitski [15,16] (i.e., the multiple deficits approach) in which frailty is viewed in terms of the number of health deficits that manifest in the individual, leading to a continuous measure of frailty (Frailty Index (FI)).

The prevalence of frailty in different populations has been widely studied. Depending on how frailty is measured, prevalence can range from 4.0% in the United States to 59.1% in the Netherlands for those aged 65 and above [17,18,19,20,21] and 13.0% in the United States to 52.0% in Australia for those aged 85 and above [8,11,22,23]. Different rates of frailty across populations suggest that the contributory factors of frailty that are culturally unique may also be different across populations. However, comparative studies of the levels of frailty across populations are sparse; although in a previous study using the Survey of Health, Ageing and Retirement in Europe (SHARE) and the study on global ageing and adult health (SAGE), frailty was reported as a useful construct that was associated with social support and healthcare systems [24]. 

In-depth cross-cultural comparisons of frailty could provide a better understanding of levels in the health of older adults, providing useful data to examine the role of personal, social and environmental factors in contributing to disparities in frailty, as well as to inform government policies. In this study, we compared the prevalence of frailty and its contributory factors across three Chinese populations: Hong Kong, Taiwan-urban and Taiwan-rural. In addition, the ratio of FI to life expectancy at birth (FI/LE) was used as an indicator of compression of morbidity for comparison across the three populations.

## 2. Materials and Methods

### 2.1. Data Sources

This is a comparison of cohorts of Hong Kong and Taiwan using two sets of aggregated secondary data on frailty prevalence and contributory factors. Since data were aggregated at the group level, relationships at the individual level were not determined.

In Hong Kong, 4000 Chinese adults aged 65 years and above were recruited by placing advertisements in housing estates and community centers, as part of a bone health survey (Osteoporotic Fractures in Men (MrOs) and Women (MsOs) (Hong Kong) Study), which started in August 2001–December 2003, with follow-up studies administered in 2003–2005 and 2005–2007. The 14-year follow-up is ongoing. The sample was stratified to recruit approximately the same number of people in each of the three age strata: 65–74, 75–84, 85+ years. Those who were unable to walk independently, had bilateral hip replacement or were not competent to give informed consent were excluded. Eligible persons were invited to attend a health check at the School of Public Health, The Chinese University of Hong Kong. A team of trained research assistants administered the study questionnaire and took physical measurements for each participant on the same day. To be comparable to the Taiwan dataset, this analysis reported results based on data from the baseline (2001–2003) only. Details of the survey population have been reported elsewhere [25]. Ethical approval was obtained from the Clinical Research Ethics Committee of the Chinese University of Hong Kong and Hospital Authority New Territories East Cluster (CRE-2003.102).

In Taiwan, 4049 individuals aged 60 years and above from the household registration population were recruited for the Taiwan Longitudinal Study on Aging (TLSA), a nationally-representative survey of adults aged 60 and above, which started in 1989, with follow-up surveys administered in 1993, 1996, 1999, 2003, 2007 and 2011. A three-stage systematic random sampling method was used for the selection of an equal probability sample. First, 56 townships and districts were selected from 331 townships and districts in Taiwan. Second, villages and neighborhoods were selected. Third, two individuals aged 60 years and above were enrolled from each selected village and neighborhood. Data were collected with face-to-face interview questionnaires by trained interviewers. To be comparable to the Hong Kong dataset, this analysis reported results based on data from the 2003 follow-up study only. In this study, after excluding those who were living in long-term care institutions and having an unknown living area, a sample of 2392 older adults aged 65 years and above was used. Detailed information of the study methodology and TLSA data collection is provided by the Health Promotion Administration of Ministry of Health and Welfare in Taiwan [26]. Research ethics committee approval was obtained from the National Cheng Kung University (A-ER-105-149).

### 2.2. Frailty Index Construction

The multiple deficits approach was used to construct the FI, which was constructed from 30 items covering self-reported medical and drug histories (14 items), functional assessments and psychological well-being (5 items), as well as geriatric syndromes (11 items). There were some variations in the definitions of some items between the Hong Kong cohort and the Taiwanese cohorts. For example, in the Hong Kong cohort, cognitive function was assessed using Mini-Mental State Examination (MMSE) [27]; depression was assessed using the 15-item Geriatric Depression Scale (GDS) [28]. In the Taiwanese cohorts, these were assessed using the Short Portable Mental Status Questionnaire (SPMSQ) [29] and the Center for Epidemiologic Studies Depression Scale (CES-D) [30], respectively. Details of the measurement methods of assessing the items are described in Appendix A Table A1. All items for FI had less than 5% missing values and were dichotomized into the presence or absence of a frailty marker, and a score of 1 representing a deficit was given to each item. The FI was calculated as the proportion of the number of deficits for an individual to the maximum total number of deficits. A cut-off point of ≥0.25 was used to indicate frailty [11,16].

### 2.3. Potential Contributory Factors

To examine factors that may contribute to frailty that are common to both datasets, the following variables were included in the analysis: basic socio-demographic characteristics including age, sex, educational attainments and living arrangement (living alone vs. not living alone) as well as lifestyle factors including smoking (current and ex-smokers vs. never smokers), alcohol use (drinkers (consumed >12 alcoholic drinks in the past 12 months) vs. non-drinkers (consumed ≤12 alcoholic drinks in past 12 months)) and physical activity (low levels (exercise <5–7 days/week or exercise <6 times/week) vs. high levels (exercise ≥5–7 days/week or exercise ≥6 times/week)).

### 2.4. Statistical Analysis

Four thousand Hong Kong men and women aged 65 years and above and 2392 Taiwanese men and women aged 65 years and above were included in this analysis. To control for the differences in age distributions across the three study populations (Hong Kong, Taiwan-urban and Taiwan-rural), standardization was applied to each of the populations using the sex and age (5-year age groups) proportions according to the 2011 Hong Kong Census Population. All analyses were performed after standardization. After standardization, the age distribution of the three populations being compared followed the same distribution as the 2011 Hong Kong Census Population. Population characteristics of Hong Kong and Taiwan were compared using analysis of variance (ANOVA) for continuous variables or the chi-square test for categorical variables. Analyses were repeated by stratifying age groups of 65–74, 75–84 and 85+ years. Prevalence of frailty was compared in each cohort for men and women, controlling for age, by the chi-square test. In addition, a ratio of the frailty index (FI) to life expectancy at birth (FI/LE) was used as an indicator of the compression of morbidity. Lower values (i.e., low FI and high LE) indicate morbidity compression. The LE is cohort- and sex-specific. In 2003, the average LE was 78.5 years for Hong Kong men and 84.4 years for Hong Kong women [31]; and 74.8 years for Taiwanese men and 80.3 years for Taiwanese women [32]. FI/LE was calculated for each participant by dividing the FI of each participant by the cohort- and sex-specific LE. Differences of FI/LE between the three populations were examined by ANOVA. This indicator has been used in a previous study [14]. Risk factors were compared between the three cohorts using crude odds ratio (OR). Variables with *p*-value < 0.1 were included in multiple logistic regressions. The dependent variable was frailty. Independent variables were age group, education level, living arrangement, smoking, alcohol use and physical activity. Attributable fractions (AF) for risk factors contributing to frailty were then compared. The AF is the proportion of the incidence of a disease in the exposed that is due to the exposure. It is the proportion of the incidence of a disease in the exposed that would be eliminated if exposure were eliminated. The AF was calculated using the formula (AF = (OR − 1)/OR)) [33]. *t*-tests were used for comparing mean continuous variables, log OR of frailty and Area Under the Curve (AUC) in logistic regression. Chi-square tests were used for comparing categorical variables. Missing observations were excluded in the analysis. Statistical analyses were performed using the Window-based SPSS Statistical Package (Version 23.0; SPSS Inc., Chicago, IL, USA). All statistical tests were two-sided. A *p*-value of < 0.05 was considered statistically significant.

## 3. Results

The FI was constructed from 30 items, and the prevalence of each was sorted into categories (medical and drug history, functional assessment and psychological well-being and geriatric syndrome) (Table 1). With respect to medical and drug history, both Taiwan-urban and -rural populations had higher prevalence rates compared to the Hong Kong population, especially in arthritis, gastropathy/gastrectomy, heart diseases, Chronic Obstructive Pulmonary Disease (COPD) and kidney disease, with the prevalence in Taiwan approximately twice that of Hong Kong. Hypertension and cataract were the top two highest prevalences of chronic disease among the three populations. The Taiwan-urban and -rural populations consumed more drugs than the Hong Kong population.

With respect to functional assessment and psychological well-being, the Taiwan-urban and -rural populations had more functional limitations (low lower limb strength and poor walking performance), but lower prevalence of cognitive impairment. In addition, the prevalence of depression was three-times higher in the Taiwan-urban and -rural populations than the Hong Kong population. With respect to geriatric syndrome, the top three syndromes with the highest prevalence showed very different patterns between the two regions. The Taiwan-urban and -rural populations had poor health and difficulty in moderate activities and climbing several stairs; however, the Hong Kong population had back pain, higher risk of falls and less activity.

There were significant differences in socio-demographic characteristics and lifestyle between the three groups (Table 2). Overall, the population characteristics of Taiwan-rural significantly differed from those of Hong Kong. The highest percentage of low education occurred in the Taiwan-rural population, followed by Hong Kong and then Taiwan-urban. Living alone was the commonest among Hong Kong women, followed by Taiwanese rural men, with the lowest percentage among men in Hong Kong. Compared to Hong Kong men, Taiwanese men had a higher percentage of smoking. An opposite pattern was observed for women. The percentage of alcohol use was the highest among Taiwanese rural men, while the percentages for Taiwanese urban men and Hong Kong men were similar. Taiwanese urban men were more active compared with Hong Kong men. However, Taiwanese women were less active compared with Hong Kong women. These differences remained significance across different age groups (Appendix A Table A2, Table A3 and Table A4).

The prevalence of frailty increased with age in all three populations and approximately doubled for every 10 years until around age 85, and the Hong Kong cohort had the lowest prevalence rate (Table 3). Overall, the prevalence of frailty was higher in women (22.6–49.7%) than in men (10.5–27.5%), and the differences were found in all age groups (*p* < 0.05). Using the ratio of FI divided by LE (i.e., FI/LE) as an indicator of the compression of morbidity, the highest ratio was observed in the Taiwan populations, with the Hong Kong population having the lowest ratio (Table 3). Similarly, the ratio was higher in women than in men in all age groups.

Risk factors for frailty were similar in all three populations (Table 4a); those having the highest odds ratios (ORs) were older, women, had low levels of physical activity and had a low educational level. Smoking and alcohol use were significantly associated with a lower risk of frailty in all three populations. Living alone was only significant in the Hong Kong cohort. In multiple logistic models, variations in the ORs and their magnitude were noted between the three populations. The highest ORs occurred in the Hong Kong cohort, for the following risk factors: older age, being a woman, low levels of physical activity and smoking. For the Taiwanese cohorts, older age, being a woman, low levels of physical activity and a low educational level (Taiwan-urban only) were significant risk factors. The risk of frailty among the older age group (aged 85+) and those who had low levels of physical activity was significantly different between the Hong Kong cohort and the Taiwanese cohorts. However, living alone was not associated with frailty in any of the three populations after controlling for covariates.

Furthermore, sex-specific analyses were performed (Table 4b,c). Except for living alone, risk factors for frailty were the same between Hong Kong and Taiwanese rural male populations. Alcohol use was inversely associated with frailty. In multiple logistic models, among Hong Kong men, the highest ORs were observed in older people, followed by those living alone and with low levels of physical activity. In Taiwanese men, older age and low levels of physical activity (Taiwan-rural only) were significant risk factors. Alcohol use was associated with a lower risk of frailty in the Hong Kong and the Taiwan-urban populations only. Among women populations, while older age (75–84) and low levels of physical activity were significant risk factors, living alone was not significant at all. In multiple logistic models, a similar pattern was observed in Hong Kong women, except that living alone was associated with a lower risk of frailty. However, living alone was not significant in Taiwanese women.

AF for frailty in the three populations are shown in Table 5. For all three populations, older age and women constituted the highest AF, while alcohol was inversely associated with frailty. AF of older age (85+) between the Hong Kong cohort and the Taiwan-urban cohort and AF of alcohol use between the Taiwan-urban and the Taiwan-rural cohorts differed significantly.

## 4. Discussion

This study compared the prevalence of frailty and its contributory factors in samples of older Chinese adults in Hong Kong and Taiwan. With a harmonized assessment of frailty, the results showed significantly different patterns. While the prevalence of frailty for Hong Kong was much lower than that in the Taiwanese cohorts, the prevalence of frailty for the Taiwanese urban population was similar to that for the Taiwanese rural population. For all cohorts, FI increased with age and was higher amongst women. However, differences in risk factors in terms of socio-demographic characteristics and lifestyle and the AF to frailty were observed across the three populations. These differences may partly explain the variations in the prevalence of frailty between studies.

With respect to socio-demographic factors, the comparison shows that there was an association between low education and frailty for Taiwanese urban women. In addition, this study found that living alone was positively associated with frailty in men, but was inversely associated with frailty in women in the Hong Kong cohort. The differences between men and women could be due to differences between the two sexes in terms of social support networks. It has been suggested that women tend to have larger and more diverse social networks than men [34,35]; therefore, women may be more able to adapt to living alone compared to men. Some studies have suggested that upon losing a spouse, men had greater mortality risk in widowhood compared with women [34,36]. In Hong Kong, living alone is a common phenomenon among older populations (Year 1991, 11.6%; Year 2014, 14.8%) [37]. The increasing number of people who live alone will probably increase the number of people with frailty. Hence, these findings emphasize the importance of social support networks in the prevention of frailty. However, the association between living arrangement and frailty was less strong in the Taiwanese cohorts compared with the Hong Kong cohort. Further research is certainly needed to fully explore whether culture differences lead to disparities in frailty.

With respect to lifestyle factors, we found that alcohol consumption was associated with lower risk of frailty in all studied cohorts. Individuals who consumed >12 alcoholic drinks in the past 12 months were less frail than those who consumed ≤12 alcoholic drinks in past 12 months. The positive association observed could be due to the abstainer/quitter bias as individuals with poor health, particularly older people, drink less than those individuals with good health. Nevertheless, previous studies showed that moderate alcohol consumption was associated with less frailty [38,39,40], possibly through anti-inflammatory mechanisms. A number of studies have also demonstrated an association between inflammatory markers (e.g., Interleukin 6 (IL-6) and C-Reactive Protein (CRP)) and prevalent frailty [41,42,43]. Unfortunately, the present study did not collect information regarding inflammatory markers. This area needs further studies and has important public health implications. As expected, there was an association between low levels of physical activity and frailty in the Hong Kong and the Taiwanese cohorts. This finding reaffirms the importance of physical activity in healthy ageing and retarding the onset of frailty [44].

Our findings also showed that functional limitations (e.g., ‘low lower limb strength’, ‘poor walking performance’) and some geriatric syndromes (e.g., ‘difficulty in moderate activities’, ‘difficulty in climbing several stairs’) were more prevalent in the Taiwanese cohorts compared to the Hong Kong cohort. The differences could be due to the use of self-reported data in the Taiwanese cohorts versus the use of objective measures (e.g., chair stands, gait speed) in the Hong Kong cohort. The Taiwanese cohorts also had a higher prevalence of depressive symptoms compared with the Hong Kong cohort, although the difference in depressive symptoms may be related to differences in the measurement used (the GDS vs. the CES-D). The higher prevalence of depressive symptoms for the Taiwanese populations suggests that there is a need to focus on measures to prevent geriatric depression. For example, screening for depression as a routine psychiatric prevention strategy is recommended. The Hong Kong cohort, on the contrary, had higher prevalence of hypertension, osteoporosis, back pain, risk of falls and fractures compared with the Taiwanese cohorts. The higher prevalence of hypertension in the Hong Kong cohort might be due, in part, to the inclusion of objective blood pressure measurement data. The Hong Kong cohort also had a high prevalence of cognitive impairment, supporting the need for including cognitive assessments in primary care settings.

In this study, we used the FI/LE ratio as an indicator of the compression of morbidity. The higher ratio for the Taiwanese cohorts compared with the Hong Kong cohort suggests that population ageing in Taiwan is projected to be accompanied by increasing frailty. This finding allows a quick comparison of the compression of morbidity between populations and provides useful data to inform government policies for health and social care planning. Frailty interventions focusing on physical activity, nutrition and cognitive impairment [45,46,47], coupled with early detection, should be developed to combat the increasing rates of frailty.

There are limitations to this study. It is a secondary comparison of cohorts from Hong Kong and Taiwan using two different datasets with data collected at different time points. In the construction of the FI, the operative definitions of some FI components differ between Hong Kong and Taiwan. Nevertheless, it has been suggested that as long as the FIs cover a range of health deficits, they could yield comparable results even though their components are not the same [48,49,50,51]. The sampling strategies are different between cohorts. In Hong Kong, a non-probability sample was used, in which subjects were more educated, more physically active and may be healthier than the general elderly population of Hong Kong. In Taiwan, an equal probability sample was used in which subjects were recruited by a multi-stage stratified random sampling method; therefore, the different strategies might have recruited older adults with different characteristics, which may have biased the impact of socioeconomic status, living arrangement and lifestyle factors on frailty. In addition, questions and definitions used in both studies varied slightly, which might lead to discrepancies. For example, in the Hong Kong cohort, low levels of physical activity were defined as less than five days of exercise per week; in the Taiwanese cohorts, it was defined as less than six times of exercise per week. These differences need to be considered in interpreting the findings. Nevertheless, this comparative study was conducted within a framework of comparable measurements, which provides useful information for the analysis of policy impact compared to studies of single countries/regions. The inclusion of performance measures of functioning in the FI, in addition to the presence of specific diseases and a set of geriatric syndromes, would also provide a basis for descriptions of health states and for undertaking comparisons of health states across populations.

## 5. Conclusions

This cross-cultural study allows us to compare the prevalence of frailty and its contributory factors in two different settings, which will enhance our understanding of variations in the underlying dynamics of population ageing in Hong Kong and Taiwan. These findings will assist policymakers of the two regions in the planning of health and social care services. This study supports the need for prevention and early detection of frailty to enable older people to achieve healthy and active ageing. Further research is needed to explore cross-cultural differences in the trajectories of frailty and its application in predicting adverse health outcomes.

## Figures and Tables

**Table 1:** Components of the frailty index.

Component	Prevalence, *n* (%)					
	Hong Kong		Taiwan-Urban		Taiwan-Rural	
	*n*	%	*n*	%	*n*	%
Medical and drug history						
Hypertension	2316	57.90	438	45.49	560	39.18
Cataract	1789	44.73	466	48.42	536	37.50
Arthritis	545	13.63	297	30.89	367	25.66
Gastropathy/gastrectomy	334	8.35	206	21.38	319	22.33
Heart diseases	401	10.03	267	27.68	318	22.24
Osteoporosis	1361	34.03	267	27.69	300	21.01
Diabetes type I or II	583	14.58	182	18.91	217	15.21
COPD	354	8.85	135	14.04	208	14.58
Gout	380	9.50	114	11.88	151	10.54
Kidney disease	165	4.13	99	10.27	139	9.76
Stroke	171	4.28	66	6.82	93	6.52
Glaucoma	200	5.00	43	4.47	85	5.94
Cancer/malignant tumor	174	4.35	39	4.02	33	2.31
Medication use (use of stimulants, sedatives, aspirin and painkillers for arthritis)	603	15.08	213	22.15	275	19.26
Functional assessment and psychological well-being						
Low lower limb strength	990	24.75	400	41.51	647	45.24
Depression	425	10.63	375	38.98	556	38.88
Poor walking performance	576	14.40	235	24.40	430	30.09
Cognitive impairment	1193	29.83	58	6.07	168	11.79
Low grip strength	498	12.45	77	7.99	141	9.89
Geriatric syndrome						
Difficulty in moderate activities	208	5.20	554	57.52	864	60.48
Difficulty in climbing several stairs	275	6.88	278	28.84	553	38.71
Poor health	275	6.88	303	31.42	552	38.62
Often feel fatigue or tired	174	4.35	246	25.51	387	27.07
Fall in past 12 months	262	6.55	188	19.53	281	19.64
Risk of falls	1092	27.30	133	13.83	260	18.19
Difficulty in movement	638	15.95	105	10.89	141	9.90
Less activity	723	18.08	78	8.06	137	9.06
Underweight	286	7.15	56	5.57	81	5.64
Ever fracture	717	17.93	38	3.97	56	3.93
Back pain	1913	47.83	27	2.82	48	3.35

**Table 2:** Population characteristics between Hong Kong, Taiwan-urban and Taiwan-rural by sex.

Characteristic	Mean (SD)/*n* (%)		
	Hong Kong	Taiwan-Urban	Taiwan-Rural
Men	*n* = 2000	*n* = 523	*n* = 742
Age, mean (SD)	74.40 (6.38)	74.51 (6.62)	74.55 (6.69)
Low education, %	1227 (61.36)	258 (49.33) ^3^	572 (77.09) ^1,2^
Living alone, %	118 (5.92)	41 (7.82)	97 (13.07) ^1,2^
Smoking, %	1303 (65.16)	355 (67.88)	568 (76.55) ^1,2^
Alcohol use, %	431 (21.56)	124 (23.71)	205 (27.63) ^1^
Low levels of physical activity, %	805 (40.24)	186 (35.56)	340 (45.82) ^1,2^
Women	*n* = 2000	*n* = 440	*n* = 687
Age, mean (SD)	76.02 (7.08)	76.08 (7.22)	76.27 (7.40)
Low education, %	1695 (84.75)	368 (83.64)	655 (95.34) ^1,2^
Living alone, %	401 (20.04)	47 (10.68) ^3^	86 (12.50) ^1^
Smoking, %	245 (12.24)	29 (6.59) ^3^	30 (4.37) ^1^
Alcohol use, %	38 (1.89)	28 (6.36) ^3^	24 (3.49) ^1,2^
Low levels of physical activity, %	645 (32.23)	234 (53.18) ^3^	379 (55.17) ^1^

^1^ *p*-value < 0.05, comparing Taiwan-rural with Hong Kong; ^2^ *p*-value < 0.05, comparing Taiwan-rural with Taiwan-urban; ^3^ *p*-value < 0.05, comparing Hong Kong with Taiwan-urban. SD: Standard Deviation.

**Table 3:** Prevalence of frailty and mean of frailty index (FI)/life expectancy at birth (LE) in different areas by age and sex.

Characteristic	Prevalence of Frailty (FI ≥ 0.25), *n* (%)			Mean (SD) of FI/LE Ratio		
	Hong Kong	Taiwan-Urban	Taiwan-Rural	Hong Kong	Taiwan-Urban	Taiwan-Rural
Men						
65–74	70 (6.43)	53 (18.66) ^3^	88 (21.89) ^1^	0.15 (0.09)	0.19 (0.16) ^3^	0.20 (0.17) ^1^
75–84	102 (14.05)	48 (25.26) ^3^	83 (31.00) ^1^	0.20 (0.13)	0.24 (0.17) ^3^	0.25 (0.17) ^1^
85+	39 (20.93)	17 (36.96) ^3^	32 (46.38) ^1^	0.24 (0.24)	0.32 (0.17) ^3^	0.31 (0.20) ^1^
Total	211 (10.54)	118 (22.69) ^3^	203 (27.51) ^1^	0.17 (0.11)	0.23 (0.16)^3^	0.23 (0.17) ^1^
Women						
65–74	134 (14.86)	69 (35.03) ^3^	124 (40.52) ^1^	0.19 (0.09)	0.27 (0.17) ^3^	0.26 (0.17) ^1^
75–84	219 (28.75)	97 (57.74) ^3^	146 (55.73) ^1^	0.25 (0.13)	0.35 (0.16) ^3^	0.34 (0.17) ^1^
85+	99 (29.31)	33 (44.59) ^3^	35 (61.32) ^1^	0.26 (0.29)	0.35 (0.19) ^3^	0.36 (0.14) ^1^
Total	452 (22.59)	199 (45.33) ^3^	335 (49.70) ^1^	0.23 (0.11)	0.32 (0.17) ^3^	0.31 (0.17) ^1^
Both sexes						
65–74	204 (10.25)	122 (25.36) ^3^	212 (29.94) ^1^	0.17 (0.09)	0.23 (0.17) ^3^	0.23 (0.18) ^1^
75–84	322 (21.58)	145 (40.50) ^3^	229 (43.29) ^1^	0.22 (0.13)	0.30 (0.18) ^3^	0.30 (0.18) ^1^
85+	138 (26.33)	50 (41.67) ^3^	97 (55.43) ^1,2^	0.25 (0.27)	0.35 (0.18) ^3^	0.35 (0.17) ^1^
Total	663 (16.57)	317 (33.06) ^3^	538 (38.10) ^1,2^	0.20 (0.12)	0.27 (0.18) ^3^	0.27 (0.18) ^1^

^1^ *p*-value < 0.05, comparing Taiwan-rural with Hong Kong; ^2^ *p*-value < 0.05, comparing Taiwan-rural with Taiwan-urban; ^3^ *p*-value < 0.05, comparing Hong Kong with Taiwan-urban. SD: Standard Deviation; FI: Frailty Index; LE: Life Expectancy.

**Table:** (**a**)

Characteristic	Crude OR (95% CI)			Adjusted OR (95% CI)		
	Hong Kong	Taiwan-Urban	Taiwan-Rural	Hong Kong	Taiwan-Urban	Taiwan-Rural
Women	2.48 (2.07, 2.96)	2.81 (2.13, 3.71)	2.60 (2.09, 3.25)	2.43 (1.92, 3.06)	2.41 (1.61, 3.60)	2.11 (1.49, 3.00)
Age						
65–74	Reference	Reference	Reference	Reference	Reference	Reference
75–84	2.41 (1.99, 2.92)	1.99 (1.48, 2.67)	1.79 (1.41, 2.26)	2.22 (1.83, 2.71)	1.92 (1.40, 2.63)	1.75 (1.37, 2.24)
85+	3.13 (2.46, 3.99)	2.06 (1.36, 3.13)	2.88 (2.05, 4.04)	2.60 (2.02, 3.34)	1.47 (0.94, 2.28) ^3^	2.38 (1.67, 3.39)
Low education	1.77 (1.43, 2.18)	2.09 (1.55, 2.83)	2.05 (1.46, 2.87)	1.23 (0.98, 1.54)	1.52 (1.09, 2.13)	1.26 (0.87, 1.82)
Smoking	0.77 (0.64, 0.91)	0.60 (0.45, 0.80)	0.48 (0.38, 0.60) ^1^	1.26 (1.01, 1.58)	1.38 (0.92, 2.09)	0.95 (0.67, 1.34)
Alcohol use	0.30 (0.20, 0.44)	0.32 (0.20, 0.51)	0.48 (0.35, 0.67)	0.50 (0.34, 0.76)	0.44 (0.27, 0.72)	0.82 (0.57, 1.16) ^2^
Low levels of physical activity	1.37 (1.16, 1.62)	2.38 (1.81, 3.13) ^3^	2.45 (1.96, 3.05) ^1^	1.51 (1.27, 1.81)	2.03 (1.52, 2.71)	2.29 (1.82, 2.88) ^1^
Living alone	1.34 (1.06, 1.69)	0.88 (0.55, 1.42)	1.02 (0.74, 1.41)	0.88 (0.69, 1.12)	0.77 (0.46, 1.28)	1.02 (0.72, 1.43)
AUC				0.679	0.703	0.700

**Table:** (**b**)

Characteristic	Crude OR (95% CI)			Adjusted OR (95% CI)		
	Hong Kong	Taiwan-Urban	Taiwan-Rural	Hong Kong	Taiwan-Urban	Taiwan-Rural
Age						
65–74	Reference	Reference	Reference	Reference	Reference	Reference
75–84	2.38 (1.73, 3.28)	1.45 (0.93, 2.26)	1.62 (1.14, 2.30)	2.05 (1.48, 2.84)	1.36 (0.86, 2.14)	1.63 (1.14, 2.33)
85+	3.86 (2.51, 5.92)	2.55 (1.31, 4.94)	3.04 (1.79, 5.16)	3.18 (2.04, 4.96)	2.25 (1.14, 4.44)	2.71 (1.58, 4.65)
Low education	1.50 (1.10, 2.04)	1.27 (0.84, 1.92)	1.52 (1.01, 2.29)	1.35 (0.98, 1.86)	1.20 (0.78, 1.83)	1.38 (0.91, 2.11)
Smoking	1.31 (0.96, 1.79)	1.19 (0.76, 1.86)	0.96 (0.65, 1.40)	1.15 (0.83, 1.59)	1.27 (0.80, 2.02)	0.96 (0.65, 1.43)
Alcohol use	0.50 (0.33, 0.76)	0.48 (0.27, 0.83)	0.67 (0.46, 0.97)	0.57 (0.37, 0.88)	0.48 (0.27, 0.86)	0.73 (0.49, 1.08)
Low levels of physical activity	1.48 (1.11, 1.97)	1.56 (1.03, 2.37)	1.96 (1.41, 2.72)	1.47 (1.10, 1.98)	1.49 (0.97, 2.28)	1.87 (1.33, 2.61)
Living alone	2.85 (1.82, 4.48)	1.00 (0.47, 2.15) ^3^	1.23 (0.77, 1.97) ^1^	2.32 (1.45, 3.71)	0.96 (0.44, 2.12)	1.21 (0.75, 1.96)
AUC				0.647	0.617	0.622

**Table:** (**c**)

Characteristic	Crude OR (95% CI)			Adjusted OR (95% CI)		
	Hong Kong	Taiwan-Urban	Taiwan-Rural	Hong Kong	Taiwan-Urban	Taiwan-Rural
Age						
65–74	Reference	Reference	Reference	Reference	Reference	Reference
75–84	2.31 (1.82, 2.94)	2.54 (1.66, 3.88)	1.84 (1.32, 2.58)	2.38 (1.86, 3.06)	2.66 (1.69, 4.18)	1.88 (1.33, 2.66)
85+	2.38 (1.76, 3.20)	1.46 (0.84, 2.51)	2.31 (1.47, 3.62)	2.30 (1.69, 3.12)	1.14 (0.64, 2.02) ^3^	2.27 (1.42, 3.63)
Low education	1.28 (0.94, 1.74)	1.99 (1.16, 3.40)	1.34 (0.65, 2.73)	1.18 (0.86, 1.62)	2.25 (1.26, 4.01)	0.96 (0.45, 2.05)
Smoking	1.58 (1.17, 2.12)	1.91 (0.88, 4.15)	0.89 (0.43, 1.85)	1.35 (0.99, 1.83)	2.35 (0.95, 5.85)	0.87 (0.40, 1.90)
Alcohol use	0.19 (0.05, 0.79)	0.32 (0.13, 0.79)	1.02 (0.45, 2.30) ^1^	0.22 (0.05, 0.92)	0.31 (0.11, 0.89)	1.43 (0.60, 3.39) ^1,2^
Low levels of physical activity	1.51 (1.22, 1.88)	2.69 (1.82, 3.97) ^3^	2.77 (2.03, 3.79) ^1^	1.55 (1.24, 1.94)	2.72 (1.80, 4.12) ^3^	2.74 (1.99, 3.78) ^1^
Living alone	0.79 (0.60, 1.04)	0.70 (0.37, 1.30)	0.88 (0.56, 1.39)	0.66 (0.50, 0.87)	0.72 (0.36, 1.42)	0.87 (0.54, 1.41)
AUC				0.629	0.695 ^3^	0.663

^1^ *p*-value < 0.05, comparing Taiwan-rural with Hong Kong; ^2^ *p*-value < 0.05, comparing Taiwan-rural with Taiwan-urban; ^3^ *p*-value < 0.05, comparing Hong Kong with Taiwan-urban. OR: Odds Ratio; AUC: Area Under the Curve.

**Table 5:** Attributable fraction for frailty in Hong Kong, Taiwan-urban and Taiwan-rural (both sexes).

Characteristic	Attributable Fraction (%) *		
	Hong Kong	Taiwan-Urban	Taiwan-Rural
Women	58.85%	58.51%	52.61%
Age			
65–74	Reference	Reference	Reference
75–84	54.95%	47.92%	42.86%
85+	61.54%	31.97% ^3^	57.98%
Low education	18.70%	34.21%	20.63%
Smoking	20.63%	27.54%	−5.26%
Alcohol use	−100.00%	−127.27%	−21.95% ^2^
Low levels of physical activity	33.77%	50.74%	56.33% ^1^
Living alone	−13.64%	−29.87%	1.96%

* A positive value indicates that the exposure is a potential risk factor for frailty, while a negative value indicates that it is a potential protective factor. ^1^ *p*-value < 0.05, comparing Taiwan-rural with Hong Kong; ^2^ *p*-value < 0.05, comparing Taiwan-rural with Taiwan-urban; ^3^ *p*-value < 0.05, comparing Hong Kong with Taiwan-urban.

## Appendix A

**Table A1:** Calculation of the frailty index.

Component	Hong Kong		Taiwan	
	Description	Value	Description	Value
Chronic disease history				
Hypertension	Self-reported doctor-diagnosed hypertension or blood pressure ≥150/90 mmHg	0 (No), 1 (Yes)	Self-reported doctor-diagnosed high blood pressure	0 (No), 1 (Yes)
Cataract	Self-reported doctor-diagnosed cataracts	0 (No), 1 (Yes)	Self-reported doctor-diagnosed cataracts	0 (No), 1 (Yes)
Arthritis	Self-reported doctor-diagnosed arthritis	0 (No), 1 (Yes)	Self-reported doctor-diagnosed arthritis or rheumatism	0 (No), 1 (Yes)
Gastropathy/gastrectomy	Surgery to remove all or part of stomach or intestines	0 (No), 1 (Yes)	Self-reported doctor-diagnosed gastric ulcer or stomach ailment	0 (No), 1 (Yes)
Heart diseases	Self-reported doctor-diagnosed heart attack, coronary or myocardial infarction	0 (No), 1 (Yes)	Self-reported doctor-diagnosed heart diseases (palpitation does not count)	0 (No), 1 (Yes)
Osteoporosis	Self-reported doctor-diagnosed osteoporosis	0 (No), 1 (Yes)	Self-reported doctor-diagnosed osteoporosis	0 (No), 1 (Yes)
Diabetes type I or II	Self-reported doctor-diagnosed diabetes	0 (No), 1 (Yes)	Self-reported doctor-diagnosed diabetes	0 (No), 1 (Yes)
COPD	Self-reported doctor-diagnosed chronic obstructive lung disease, chronic bronchitis, asthma, emphysema or COPD	0 (No), 1 (Yes)	Self-reported doctor-diagnosed bronchitis, pneumonia, asthma, pulmonary diseases or other respiratory ailment	0 (No), 1 (Yes)
Gout	Self-reported doctor-diagnosed gout	0 (No), 1 (Yes)	Self-reported doctor-diagnosed gout	0 (No), 1 (Yes)
Kidney disease	Self-reported doctor-diagnosed kidney stones	0 (No), 1 (Yes)	Self-reported doctor-diagnosed renal disease (including stone)	0 (No), 1 (Yes)
Stroke	Self-reported doctor-diagnosed stroke	0 (No), 1 (Yes)	Self-reported doctor-diagnosed stroke	0 (No), 1 (Yes)
Glaucoma	Self-reported doctor-diagnosed glaucoma	0 (No), 1 (Yes)	Self-reported glaucoma and other eye diseases (presbyopia and blindness do not count)	0 (No), 1 (Yes)
Cancer/malignant tumor	Self-reported doctor-diagnosed cancer	0 (No), 1 (Yes)	Self-reported doctor-diagnosed cancer or malignant tumor	0 (No), 1 (Yes)
Medication use	Number of medication (stimulants, sedatives, aspirin, painkillers for arthritis)	0 (No), 1 (≥1)	Number of medication (stimulants, sedatives, aspirin, painkillers for arthritis)	0 (No), 1 (≥1)
Functional assessment and psychological wellbeing				
Low lower limb strength	Repeated chair stand (seconds to complete 5 stands)	0 (≤15), 1 (>15)	Self-reported difficulty in squatting	0 (No), 1 (Yes)
Depression	GDS	0 (<8), 1 (≥8)	CES-D	0 (<8), 1 (≥8)
Poor walking performance	Six meter usual pace (minimum time to walk 6 m, seconds)	Male: 0 (≤7.65), 1 (>7.65) Female: 0 (≤8.63), 1 (>8.63)	Self-reported difficulty in walking for 200–300 m	0 (No), 1 (Yes)
Cognitive impairment	MMSE	0 (≥24), 1 (<24)	SPMSQ	0 (≥6), 1 (<5)
Low grip strength	Grip strength (max of right/left grip strength, kg)	Male: 0 (≥25), 1 (<25) Female: 0 (≥17), 1 (<17)	Self-reported difficulty in grasping or turning objects with figures	0 (No), 1 (Yes)
Geriatric syndrome				
Difficulty in moderate activities	Self-reported difficulty in moderate activities such as moving a table, pushing a vacuum cleaner	0 (No, not limited at all), 1 (Yes, limited a lot/a little)	Self-reported difficulty in running a short distance	0 (No), 1 (Yes)
Difficulty in climbing several stairs	Self-reported difficulty in climbing several flights of stairs	0 (No, not limited at all), 1 (Yes, limited a lot/a little)	Self-reported difficulty in climbing several flights	0 (No), 1 (Yes)
Poor health	Self-rated health (compared to other people of your own age)	0 (Excellent/good/fair for my age), 1 (Very poor/poor for my age)	Self-rated current health	0 (Excellent /good/fair), 1 (Very poor/poor)
Often feel fatigue or tired	Have had much energy during the past 4 weeks	0 (A good bit of/most of/all of the time), 1 (None of/a little of/some of the time)	A selected item from the SWLS (feeling most of the things done are dull (not fun))	0 (No), 1 (Yes)
Fall in the past 12 months	Have fallen and landed on the floor or fallen and hit an object like a table or a chair in the past 12 months	0 (<2 time), 1 (≥2 time)	Have ever tumbled or fallen in the past year (including a tumble during walking, slip, failure to sit well or stand firmly, or fall because of dizziness, or fall off the bed, regardless of getting injured or not)	0 (No), 1 (Yes)
Risk of fall	Have trouble with dizziness	0 (No), 1 (Yes)	Have trouble with dizziness	0 (No), 1 (Yes)
Difficulty in movement	Physical limitations in work or other regular daily activities during the past 4 weeks	0 (No), 1 (Yes)	ADLs	0 (<1), 1 (≥1)
Less activity	Less activity during the past 4 weeks	0 (No), 1 (Yes)	Less activity during the past month due to illness or injury	0 (No), 1 (≥1 day)
Underweight	BMI, kg/m^2^	0 (≥18.5 kg/m^2^), 1 (<18.5 kg/m^2^)	BMI, kg/m^2^	0 (≥18.5 kg/m^2^), 1 (<18.5 kg/m^2^)
Ever fracture	Self-reported doctor-diagnosed fracture (have broken or fractured a bone)	0 (0 time), 1 (≥1 time)	Self-reported doctor-diagnosed hipbone fracture	0 (No), 1 (Yes)
Back pain	Self-reported back pain during the past 12 months	0 (No), 1 (Yes)	Self-reported back pain	0 (No), 1 (Yes)

ADL, Activities of Daily Living; BMI, Body Mass Index; CES-D, Center for Epidemiologic Studies Depression Scale; COPD, Chronic Obstructive Pulmonary Disease; GDS, Geriatric Depression Scale; MMSE, Mini-Mental State Examination; SPMSQ, Short Portable Mental Status Questionnaire; SWLS, Satisfaction with Life Scale.

**Table A2:** Population characteristics between Hong Kong, Taiwan-urban and Taiwan-rural: age 65–74.

Characteristic	Mean (SD)/*n* (%)		
	Hong Kong	Taiwan-Urban	Taiwan-Rural
Men	*n* = 1087	*n* = 284	*n* = 403
Age, mean (SD)	69.50 (2.37)	69.62 (3.24)	69.53 (2.94)
Low education, %	620 (57.07)	139 (48.77) ^3^	308 (76.43) ^1,2^
Living alone, %	37 (3.41)	16 (5.61)	48 (11.91) ^1,2^
Smoking, %	662 (60.87)	183 (64.44)	312 (77.23) ^1,2^
Alcohol use, %	293 (27.00)	80 (28.07)	128 (31.68)
Low levels of physical activity, %	420 (38.63)	100 (35.21)	185 (45.91) ^1,2^
Women	*n* = 900	*n* = 198	*n* = 309
Age, mean (SD)	69.44 (2.17)	69.47 (2.92)	69.54 (3.02)
Low education, %	724 (80.38)	166 (83.84)	290 (93.85) ^1,2^
Living alone, %	107 (11.86)	17 (8.59)	32 (10.36)
Smoking, %	62 (6.91)	7 (3.54)	10 (3.24) ^1^
Alcohol use, %	29 (3.22)	14 (7.07) ^3^	16 (5.18)
Low levels of physical activity, %	298 (33.12)	97 (48.99) ^3^	160 (51.61) ^1^

^1^ *p*-value < 0.05, comparing Taiwan-rural with Hong Kong; ^2^ *p*-value < 0.05, comparing Taiwan-rural with Taiwan-urban; ^3^ *p*-value < 0.05, comparing Hong Kong with Taiwan-urban. SD: Standard Deviation.

**Table A3:** Population characteristics between Hong Kong, Taiwan-urban and Taiwan-rural: age 75–84.

Characteristic	Mean (SD)/*n* (%)		
	Hong Kong	Taiwan-Urban	Taiwan-Rural
Men	*n* = 727	*n* = 190	*n* = 270
Age, mean (SD)	78.48 (2.86)	78.45 (2.76)	78.61 (2.70)
Low education, %	486 (66.80)	85 (44.74) ^3^	208 (77.04) ^1,2^
Living alone, %	51 (7.03)	23 (12.11) ^3^	38 (14.07) ^1^
Smoking, %	512 (70.40)	139 (73.16)	208 (77.04) ^1^
Alcohol use, %	116 (15.97)	36 (18.85)	65 (24.07) ^1^
Low levels of physical activity, %	324 (44.62)	66 (34.55) ^3^	113 (42.01)
Women	*n* = 763	*n* = 168	*n* = 262
Age, mean (SD)	78.94 (3.04)	78.87 (2.76)	79.01 (2.85)
Low education, %	675 (88.50)	136 (80.95) ^3^	252 (96.18) ^1,2^
Living alone, %	219 (28.64)	24 (14.29) ^3^	35 (13.36) ^1^
Smoking, %	113 (14.82)	12 (7.19) ^3^	17 (6.49) ^1^
Alcohol use, %	9 (1.15)	8 (4.76) ^3^	8 (3.04) ^1^
Low levels of physical activity, %	236 (30.94)	87 (51.79) ^3^	146 (55.51) ^1^

^1^ *p*-value < 0.05, comparing Taiwan-rural with Hong Kong; ^2^ *p*-value < 0.05, comparing Taiwan-rural with Taiwan-urban; ^3^ *p*-value < 0.05, comparing Hong Kong with Taiwan-urban. SD: Standard deviation.

**Table A4:** Population characteristics between Hong Kong, Taiwan-urban and Taiwan-rural: age 85+.

Characteristic	Mean (SD)/*n* (%)		
	Hong Kong	Taiwan-Urban	Taiwan-Rural
Men	*n* = 186	*n* = 49	*n* = 69
Age, mean (SD)	87.09 (4.51)	87.71 (2.18)	87.97 (3.08)
Low education, %	121 (65.12)	35 (71.43)	56 (81.16) ^1^
Living alone, %	30 (16.28)	2 (4.08) ^3^	11 (15.94) ^2^
Smoking, %	130 (69.77)	33 (67.35)	48 (70.59)
Alcohol use, %	22 (11.63)	9 (18.37)	13 (18.84)
Low levels of physical activity, %	60 (32.56)	21 (42.86)	41 (59.42) ^1^
Women	*n* = 337	*n* = 74	*n* = 116
Age, mean (SD)	87.00 (6.20)	87.44 (2.33)	87.90 (3.02)
Low education, %	296 (87.93)	65 (87.84)	113 (97.41) ^1,2^
Living alone, %	75 (22.41)	6 (8.11) ^3^	18 (15.52)
Smoking, %	70 (20.69)	9 (12.16)	3 (2.59) ^1,2^
Alcohol use, %	0 (0.00)	6 (8.11) ^3^	0 (0.00) ^2^
Low levels of physical activity, %	110 (32.76)	50 (67.57) ^3^	74 (64.35) ^1^

^1^ *p*-value < 0.05, comparing Taiwan-rural with Hong Kong; ^2^ *p*-value < 0.05, comparing Taiwan-rural with Taiwan-urban; ^3^ *p*-value < 0.05, comparing Hong Kong with Taiwan-urban. SD: Standard Deviation.
